# Effect of fruit and vegetable concentrates on endothelial function in metabolic syndrome: A randomized controlled trial

**DOI:** 10.1186/1475-2891-10-72

**Published:** 2011-06-29

**Authors:** Ather Ali, Yuka Yazaki, Valentine Y Njike, Yingying Ma, David L Katz

**Affiliations:** 1 Prevention Research Center, Yale University School of Medicine, Griffin Hospital, 130 Division Street, 2nd Floor, Derby, Connecticut, 06418, USA

**Keywords:** phytonutrients, dietary supplements, cardiovascular, antioxidant, randomized, fruit, vegetable

## Abstract

**Background and Objective:**

Dehydrated fruit and vegetable concentrates provide an accessible form of phytonutrient supplementation that may offer cardioprotective effects. This study assessed the effects of two blends of encapsulated juice powder concentrates (with and without added berry powders) on endothelial function in persons with metabolic syndrome, a risk factor for type 2 diabetes and cardiovascular disease.

**Methods:**

Randomized, double blind, placebo controlled crossover clinical trial with three treatment arms. 64 adults with metabolic syndrome were enrolled and received 8-week sequences of each blend of the concentrates and placebo. The primary outcome measure was change in endothelial function (assessed as flow-mediated dilatation of the brachial artery) 2 hr after consuming a 75 g glucose load, after 8-weeks of daily consumption (sustained) or 2 hr after consumption of a single dose (acute). Secondary outcome measures included plasma glucose, serum insulin, serum lipids, and body weight.

**Results:**

No significant between-group differences in endothelial function with daily treatment for 8 weeks were seen. No other significant treatment effects were discerned in glucose, insulin, lipids, and weight.

**Conclusion:**

Encapsulated fruit and vegetable juice powder concentrates did not alter insulin or glucose measures in this sample of adults with metabolic syndrome.

**Trial Registration:**

clinicaltrials.gov NCT01224743

## Background

Among non-pharmacologic approaches to cardiovascular disease prevention, regular consumption of fruit and vegetables demonstrates a dose-response effect in reducing risk for coronary heart disease [1,2]. Green leafy vegetables and foods high in vitamin C content appear to have the strongest relationship to cardiac risk reduction [1]. Dark berries have been shown to improve blood pressure, platelet function, vascular function, and lipids likely due to the relatively high levels of antioxidant polyphenols [3]. Phytonutrients in fruits and vegetables, including flavonoids [4,5] may have specific cardioprotective effects partially mediated through favorable effects on endothelial function [6].

Despite multiple public health measures designed to increase fruit and vegetable consumption [7,8], population intake levels are suboptimal [9,10]. Barriers to widespread increases in fruit and vegetable intake include cost and lack of access in medically underserved areas [7]. Dehydrated fruit and vegetable juice concentrates, available in capsule form, provide an accessible form of phytonutrient supplementation that may provide similar cardioprotective effects.

Because of the strong correspondence between peripheral and coronary endothelial responses [11], measurement of endothelial-dependent flow-mediated dilatation of the brachial artery with the use of high-resolution ultrasound scanning has become a standard research assessment method [12]. Endothelial function may be acutely impaired by consuming a glucose load [13]; therefore, various vasculoprotective [14,15] and antioxidant [16] interventions can mitigate acute endothelial dysfunction induced by hyperglycemia.

This study aimed to assess the effects of commercially available encapsulated fruit and vegetable juice powder concentrates (Juice Plus+) on post-glucose challenge endothelial function and cardiac risk markers in patients with metabolic syndrome, a constellation of risk factors directly promoting atherosclerosis [17]. We hypothesized that encapsulated fruit and vegetable concentrates can provide cardioprotective benefits similar to the effects of regular consumption of whole fruits and vegetables.

## Methods

### Ethics Statement

The study protocol and consent form were approved by the Griffin Hospital (Derby, CT) Institutional Review Board and the Yale University (New Haven, CT) Human Investigation Committee and conducted in accordance with the Declaration of Helsinki. Written informed consent was obtained, and all subjects received nominal monetary compensation for their participation.

### Participants

Subjects were female or male, 18 years of age or older with metabolic syndrome [18]. Eligible subjects were nonsmokers (as smoking impairs endothelial function [19]), not taking any other vitamins or dietary supplements, and able to have blood pressure measured bilaterally at the brachial artery. Subjects were excluded with any unstable medical condition that would limit the ability to participate fully in the trial. Subjects were also excluded if using insulin, glucose sensitizing medication, and vasoactive medications (including glucocorticoids, antineoplastic agents, psychoactive agents, or bronchodilators). Other exclusion criteria included rheumatologic disease requiring regular use of NSAIDs or alternative medications, preexisting cardiovascular disease, or a diagnosed eating disorder.

Metabolic syndrome was defined using the International Diabetes Federation criteria [18] of: waist circumference of ≥ 94 cm (males) or ≥ 80 cm (females) plus any two of the following: (a) blood pressure ≥ 130/85 or taking antihypertensive medication, (b) fasting plasma glucose (FPG) > 100 mg/dL, (c) serum triglycerides (TG) > 150 mg/dL, (d) high-density lipoprotein (HDL) < 40 mg/dL in men, and < 50 mg/dL in women. Subjects on lipid lowering medication were assumed to meet criteria for (c) and (d) above, though required to have a stable dose for at least three months and willing to refrain from taking medication for 12 hours prior to endothelial function assessment.

### Study Design and Interventions

This study was a randomized, double blind, placebo controlled crossover clinical trial with three treatment arms designed to assess the effects of 8 weeks of daily ingestion of two encapsulated fruit and vegetable juice powder concentrates (Juice Plus+) vs. placebo. The two blends were chosen to further assess whether the addition of berry powders (Blend 1) would result in differential effects compared to a general blend of fruits and vegetables (Blend 2). Blend 1 consisted of a combination of fruit, vegetable, and berry mixtures containing 7.5 mg β-carotene, 276 mg vitamin C, 72 mg vitamin E (RRR-α-tocopherol), 780 μg folate, and 80 mg calcium [20,21]. Blend 2 consisted of a combination of fruit and vegetable mixtures containing 7.5 mg β-carotene, 234 mg vitamin C, 30 mg vitamin E (RRR-α-tocopherol), 420 μg folate, and 60 mg calcium [21]. A description of the blends used in the study are in Table 1.

**Table 1:** Description of Interventions

Blend 1	Blend 2
***Consisted of juice powder concentrates containing***: Apple, Orange, Pineapple, Cranberry, Peach, Acerola cherry, Papaya, Carrot, Parsley, Beet, Kale, Broccoli, Cabbage, Spinach, Tomato *With added berry powders containing:* Concord grape, Blueberry, Cranberry, Blackberry, Bilberry, Raspberry, Red currant, Black currant, Elderberry, Green tea, Ginger root, Grape seed, Artichoke	*Consisted of juice powder concentrates containing*: Apple, Orange, Pineapple, Cranberry, Peach, Acerola cherry, Papaya, Carrot, Parsley, Beet, Kale, Broccoli, Cabbage, Spinach, Tomato

Source: Natural Standard Database: www.naturalstandard.com

**Figure 1:**
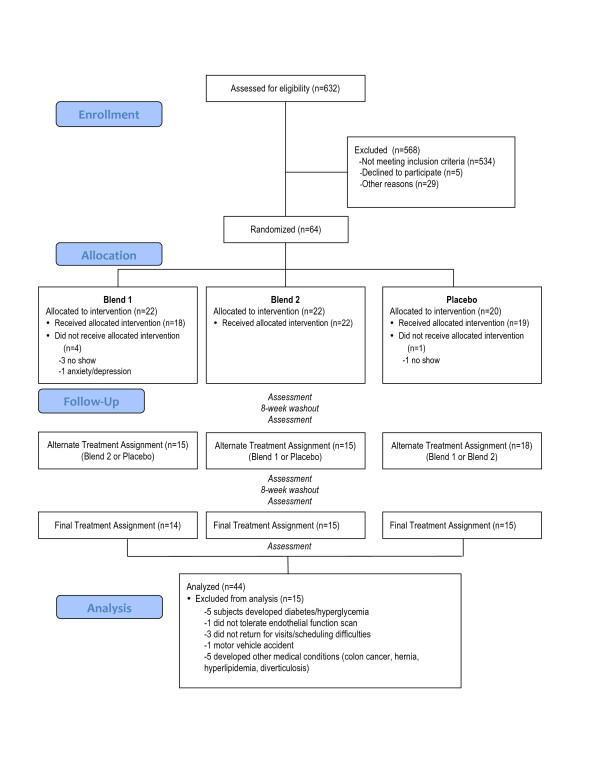
**Flow of participants in the trial**.

For the initial assignment, subjects were randomized to Blend 1, Blend 2, or placebo. As the pharmacokinetics of the tested blends are unknown, our design incorporated an 8-week washout period with no intervention after completing the initial treatment assignment. Subjects were then crossed-over to one of the two remaining assignments. Following 8-weeks of sustained intervention in the second assignment and an 8-week washout period, subjects then crossed-over to the remaining treatment assignment for 8-weeks followed by a final 8-week washout period. Subjects were evaluated on six occasions during the study: baseline, at the completion of each of the three sustained treatment assignments, and following two washout periods (see Figure 1)

Subjects were instructed to take three capsules twice daily for a total of six capsules per day (4.5 grams). Placebo capsules were provided by the study sponsor and were identical in appearance to active treatments to ensure blinding. Subjects continued consuming their usual diet; no dietary advice or guidance was provided during the course of the study.

Recruitment: Screening commenced in December 2004. Of the 632 persons screened for eligibility, 534 did not meet eligibility criteria, 5 refused to participate, and 29 were not randomized for other reasons (see Figure 1). Enrollment continued until October 2006. 64 subjects (52% female, mean age 56.9 years) were randomized to sequences of Blend 1, Blend 2, and placebo. Subjects received the intervention between July 2005 and October 2007. Of the enrolled subjects, 48 completed at least 1 time point and 44 completed the entire trial (see Figure 1). Pill count data was available for 75% of all supplement bottles dispensed; 84% of these demonstrated compliance of over 80%.

### Primary outcome measure

Endothelial function was assessed as flow-mediated dilatation (FMD) of the brachial artery using standard procedures [22]. In brief, FMD was measured noninvasively in the right brachial artery with a high-frequency ultrasound (Sonos 4500; Phillips Medical Systems, Andover, MA) in accordance with published guidelines [12]. Measures of vessel diameter and flow velocity were obtained by a single dedicated vascular clinical research specialist (YM) blinded to subject treatment status. Repeat scans were obtained at 15, 60, and 120 seconds after deflation. At each scanning interval, both cross-sectional vessel diameter and flow velocity were recorded. In addition to brachial diameter at 60 seconds after cuff release, flow after cuff deflation within the first 15 seconds was used as an indicator of stimulus strength; hyperemic flow being the stimulus for endothelial reactivity. To account for potential variability in stimulus strength, FMD was divided by flow at 15 seconds after cuff deflation to create a stimulus-adjusted response measure.

Normal FMD in young, healthy subjects is typically > 10% [23]. Impaired FMD is generally < 10%; Su et al. reported mean FMD values of 10.68 in those with normal glucose tolerance, 8.86 in patients with impaired glucose tolerance, and 5.27 in type 2 diabetes mellitus [24].

The primary outcome measures were the difference in FMD between the two blends of supplements and placebo following a 2-hour oral glucose tolerance test (OGTT) after 8 weeks of treatment.

### Secondary Outcome Measures

Change in the fasting (unchallenged) FMD after 8 weeks on active treatment and placebo assignments, the difference in FMD between Blend 1 and Blend 2, weight, lipid panel, and serum insulin. Acute (single dose) effects on endothelial function and plasma glucose were assessed by consuming the supplements concurrently with a 75 g glucose challenge load and assessing FMD after two hours.

Outcomes were assessed and laboratory measures were collected and analyzed at Griffin Hospital (Derby, CT) as routine clinical samples following each visit.

### Randomization

Subjects meeting eligibility criteria and enrolling in the study were randomized by the study sponsor to treatment sequence using the web site Randomization.com (http://www.randomization.com). The sponsor provided coded bottles for each subject containing their daily capsules. Subjects were enrolled and assigned to interventions by the study coordinator. All study personnel and subjects were blinded to treatment assignment throughout the duration of the intervention.

### Statistical Analysis

Statistical analysis was conducted using SAS software (Version 9.1, SAS Institute, Cary, NC, USA). Repeated measures ANOVA were performed to compare the means of both dependent and independent variables at baseline and after each washout period between the three treatment groups. To adjust for any potential carryover effect following washout, treatment sequence (timing of each treatment) was entered as a control variable in multivariable analysis.

Analysis was by intention-to-treat. Missing individual data was addressed by the last observation carried forward method. Data were assessed for normality prior to analysis. Consistent with guidelines from the International Brachial Artery Reactivity Task Force [12], a sample size of 48 individuals, allowing for 20% attrition and non-adherence, was predicted to provide 80% power to detect a minimal clinically important difference of 3.0% in post-glucose load FMD between intervention and placebo groups following 8 weeks of daily treatment, as well as adjusting for multiple comparisons for three pair-wise comparisons (two-tailed α = 0.05).

### Ancillary Analyses

Per-protocol analysis included subjects that completed at least one treatment assignment. Paired t-tests were used to compare baseline measures of FMD and plasma analyses to the values obtained at the end of each washout period (within-group analyses).

## Results

Baseline characteristics of the study participants are provided in Table 2. All subjects were overweight; the mean body-mass index (BMI) of participants at baseline was 31.8 kg/m^2^. Endothelial function was partially compromised with an average FMD of 8% at baseline. Subjects were pre-diabetic [25] with a mean FPG of 115 mg/dL.

**Table 2:** Baseline Values (n = 60)

Variable	Values (mean ± SD)
*Demographics*	
Female (n,%)	31 (52%)
Age	56.9 ± 11.2
*Endothelial Function*	
Flow-mediated dilatation, *%*	8.00 ± 3.70
Stimulus-adjusted response measure	0.11 ± 0.21
*Serum Measures*	
Insulin, *pmol/L*	346.56 ± 225.71
Fasting plasma glucose, *mmol/L*	6.37 ± 1.86
*Anthropometric Measures*	
Body Weight, *kg*	89.00 ± 18.80
Body Mass Index, *kg/m^2^*	31.80 ± 5.60
*Lipid Panel*	
Total Cholesterol, *mmol/L*	5.28 ± 1.00
High Density Lipoprotein (HDL), *mmol/L*	1.09 ± 0.25
Total Cholesterol/HDL	5.10 ± 1.40
Triglycerides, *mmol/L*	2.00 ± 1.16
Low Density Lipoprotein (LDL), *mmol/L*	3.33 ± 0.91

### Endothelial Function

Compared to placebo, no significant differences (*P *> 0.05) were seen in FMD change between baseline and 8-weeks in subjects consuming Blend 1 or Blend 2 (Table 3). Findings persisted after controlling for the variability of the strength of the stimulus that determines vasodilatation (Table 2). No significant deterioration in endothelial function was seen in any group after consuming the glucose load (Table 3). A random sample of 20 FMD measurements were reanalyzed; the correlation (Pearson's *r*) between the initial and second assessment values was 99%.

**Table 3:** Change in Outcome Measures after 8 weeks (Intention-to-treat analysis) (n = 55-60)

Variable	Blend 1 (mean ± SD)	Blend 2 (mean ± SD)	Placebo (mean ± SD)
*Endothelial Function*			
Flow-mediated dilatation, *%*	0.70 ± 3.70	0.70 ± 3.80	0.60 ± 3.70
Stimulus-adjusted response measure	-0.02 ± 0.23	-0.02 ± 0.21	-0.03 ± 0.21
Flow-mediated dilatation, *% (acute effects)*	1.1 ± 3.70	0.7 ± 3.5	-0.3 ± 5.4
*Serum Measures*			
Insulin, pmol/L	68.06 ± 311.83	18.06 ± 213.21	15.97 ± 202.79
Fasting plasma glucose, mmol/L	0.03 ± 1.44	0.16 ± 1.47	0.38 ± 1.58
*Anthropometric Measures*			
Body Weight, kg	0.40 ± 2.20	0.30 ± 2.80	0.40 ± 2.20
Body Mass Index, kg/m^2^	0.10 ± 0.80	0.10 ± 1.00	0.10 ± 0.80
*Lipid Panel*			
Total Cholesterol, mmol/L	0.08 ± 0.47	0.08 ± 0.60	0.03 ± 0.52
High Density Lipoprotein (HDL), mmol/L	0.04 ± 0.17	0.04 ± 0.12	0.06 ± 0.15
Total Cholesterol/HDL	-0.10 ± 0.60	-0.10 ± 0.80	-0.30 ± 0.70
Triglycerides, mmol/L	0.02 ± 0.64	-0.01 ± 0.71	-0.13 ± 0.68
Low Density Lipoprotein (LDL), mmol/L	-0.02 ± 0.53	0.08 ± 0.62	0.02 ± 0.53

p > 0.05 for all pairwise comparisons

### Serum Measures

No significant changes in serum insulin were seen in either intervention group between baseline and 8-weeks compared to placebo (*P*>0.05). Furthermore, no significant differences were seen between Blend 1, Blend, 2, and placebo in FPG and total cholesterol (all *P*>0.05) (Table 3).

### Weight

Body weight did not change in any group after eight weeks.

### Ancillary Analyses

Per-protocol analyses consisting of subjects that completed the entire intervention (n = 27-37) did not reveal any major differences from the intention-to-treat analysis. Furthermore, analyses of highly compliant subjects (>80% compliance) and the combination of compliant subjects completing the trial did not find any significant treatment effects of either blend (Table 4). Acute (single dose) effects of Blend 1 trended towards a significant improvement in endothelial function two hours after consumption of a 75-g glucose load when compared to baseline (Blend 1: 1.1 ± 3.7%, *P *= 0.0549). No significant between-group differences were found. No effects of gender or age were found.

**Table 4:** Change in Outcome Measures after 8 weeks (For subjects with at least 80% compliance) (n = 27-37)

Variable	Blend 1 (mean ± SD)	Blend 2 (mean ± SD)	Placebo (mean ± SD)
*Endothelial Function*			
Flow-mediated dilatation, *%*	0.60 ± 4.30	1.00 ± 3.40^1^	0.60 ± 4.20
Stimulus-adjusted response measure	-0.04 ± 0.33	-0.04 ± 0.28	-0.05 ± 0.28
*Serum Measures*			
Insulin, pmol/L	65.28 ± 362.53	-29.17 ± 232.66	20.14 ± 225.71
Fasting plasma glucose, mmol/L	0.16 ± 1.75	0.11 ± 1.69	0.07 ± 1.61
*Anthropometric Measures*			
Body Weight, kg	0.90 ± 2.90	0.20 ± 2.50	0.40 ± 2.00
Body Mass Index, kg/m^2^	0.30 ± 0.90	0.00 ± 0.80	0.10 ± 0.70
*Lipid Panel*			
Total Cholesterol, mmol/L	0.10 ± 0.62	0.04 ± 0.49	0.01 ± 0.62
High Density Lipoprotein (HDL), mmol/L	0.04 ± 0.21	0.06 ± 0.15	0.10 ± 0.17
Total Cholesterol/HDL	-0.10 ± 0.70	0.00 ± 0.80^1^	-0.40 ± 0.80
Triglycerides, mmol/L	0.03 ± 0.71	0.24 ± 0.60	-0.19 ± 0.87
Low Density Lipoprotein (LDL), mmol/L	-0.04 ± 0.71	0.11 ± 0.55	-0.01 ± 0.74

^1 ^p = 0.02 as compared to placebo;

### Adverse Effects

No adverse effects related to the intervention were seen during the course of the study.

## Discussion

Daily supplementation with these two blends of encapsulated fruit and vegetable juice powder concentrates did not improve endothelial function or other cardiac risk measures in this population of adults with metabolic syndrome; no significant between-group differences were seen. Contrary to expectation, acute glucose loading did not induce endothelial dysfunction. The acute administration of fruit and vegetable blends in combination (Blend 2) appeared to augment endothelial function.

Previous research in fruit and vegetable juice powder concentrates demonstrated significant improvements in serum antioxidant levels after four weeks [26] or 60 days [21] of daily supplementation. Despite compelling epidemiological [27-32] and preclinical [33,34] evidence supporting the use of antioxidant supplements, several large randomized trials [35-41] have not demonstrated significant benefits of antioxidant supplements on cardiac risk, with some possibility of increased risk [42-44]. A common hypothesis for these apparently discrepant findings is that residual confounding occurred; that is, people that use dietary supplements also tend to incorporate cardioprotective lifestyle habits such as a prudent diet and regular exercise regimens at a higher prevalence than persons who do not regularly use dietary supplements [45].

A previous trial demonstrated positive effects of four weeks of supplementation with the same phytonutrient blends used in our study on FMD in reducing the detrimental effects of a high-fat meal on FMD, assessed three hours after consumption of the meal [46]. Our subjects did not consume a high-fat meal prior to endothelial function assessment; rather they consumed a 75-g glucose load 2-hours prior to acute assessment. The literature is mixed regarding the effects of a glucose load on endothelial function; some studies have found deterioration [13,47-50] while others have found no change [51-53] in endothelial function following consumption of a glucose load. We expected that a glucose challenge load would impair endothelial function similar to the impairments seen with a high-fat meal. Our subjects, however, did not experience a significant change in endothelial function following the consumption of a 75-gram glucose load (Table 4). Thus, it is possible that acute effects of the fruit and vegetable juice powder concentrates were attenuated in light of relatively unimpaired vascular function, or that supplementation with these concentrates provided a protective effect on the vasculature attenuating any deleterious effects of the glucose load. A somewhat surprisingly normal mean baseline FMD and a lack of overt degradation in response to a glucose challenge in our subjects may have mitigated against any observable treatment effect.

It is possible that eight weeks of intervention was too short in duration to demonstrate significant sustained effects in endothelial function and cardiac risk markers. However, in our own lab, we have demonstrated significant improvements two hours after acute ingestion [22], as well as, after six weeks of daily consumption of cocoa [54] containing antioxidant flavonoids. Furthermore, a recent trial of whole fruits and vegetables (1-6 servings/day) demonstrate significant benefits in endothelial function measured by venous occlusion plethysmography in a dose-response manner in hypertensive patients, leading to a 6.2% improvement in endothelium-dependent forearm blood flow after eight weeks [55].

Whole fruits and vegetables contain significant amounts of dietary fiber, demonstrated to have cardioprotective effects [56] and the ability to reduce coronary heart disease events [2] and improve endothelial function [55]. The supplements used in this trial were encapsulated fruit and vegetable juice powder concentrates with minimal fiber. It is possible that dietary fiber mediates the beneficial effects of fruits and vegetables on endothelial function and cardiovascular risk. Furthermore, fiber in the diet promotes satiety [57,58]; thus persons adding fruits and vegetables to their routines may be displacing the consumption of other foods that promote weight gain and increase cardiac risk. The small capsules (4.5 g in six capsules) used in this study are unlikely to affect satiety and thus displace the consumption of atherogenic foods.

Strengths of this study include a crossover design increasing statistical power and a well-tolerated, commercially available intervention. Limitations of this study include the lack of antioxidant biomarkers to corroborate serum antioxidant levels with supplement consumption. It is possible that subjects with low serum antioxidant levels may benefit from supplementation more than persons with normal or supranormal levels. Other limitations include a relatively homogenous sample; largely Caucasian in a suburban community setting, as well as a lack of data regarding dietary, exercise habits, and alcohol intake that could possibly affect endothelial function.

## Conclusion

Encapsulated fruit and vegetable concentrates did not alter insulin or glucose measures. Acute endothelial dysfunction was not observed with glucose loading, mitigating against observable treatment effects. Further study with more overt impairment of endothelial function is warranted.

## Competing interests

The authors declare that they have no competing interests.

## Authors' contributions

AA wrote the manuscript and interpreted the data. YY coordinated the trial and data collection. VYN conducted the statistical analysis and provided critical review of the manuscript. YM conducted the endothelial function assessments. DLK designed and supervised the study, obtained funding, interpreted the data, and provided critical review of the manuscript. All authors have read and approved the final manuscript.
